# Evaluating the impact of “Anatomy Cluedo” on cognitive learning and game mechanics: A pilot gamification study in cranial nerve anatomy

**DOI:** 10.12669/pjms.42.1.12758

**Published:** 2026-01

**Authors:** Sarah Khalid, Rehan A Khan, M. Suleman Sadiq Hashmi, Masood Jawaid

**Affiliations:** 1 Sarah Khalid Department of Anatomy, Shalamar Medical and Dental College, Lahore, Pakistan; 2 Rehan A Khan Riphah International University, Islamabad, Pakistan; 3 M. Suleman Sadiq Hashmi Department of Medical Education, Shalamar Medical and Dental College, Lahore, Pakistan; 4 Masood Jawaid Department of Medical Affairs at PharmEvo (Pvt), Karachi, Pakistan

**Keywords:** Gamification, Anatomy, Anatomy Cluedo, Cranial Nerves

## Abstract

**Objective:**

This study aimed to evaluate the impact of the gamified learning tool “Anatomy Cluedo” on cognitive learning gain and engagement in cranial nerve anatomy among medical students.

**Methodology:**

The quantitative research study was carried out in the Department of Anatomy, Shalamar Medical and Dental College, Lahore. In this study 40 students were divided into two groups: the experimental group engaged with “Anatomy Cluedo,” while the control group participated in problem-based learning (PBL). Pre-test and post-test assessments, along with engagement metrics measured by the Flow Short Scale (FSS), were analyzed.

**Results:**

The results showed significantly higher cognitive gains in the experimental Group-A (p < 0.001), and strong positive correlations between engagement metrics and learning outcomes (r = 0.78, p < 0.01). Key game elements, such as mechanics, feedback and storytelling, were identified as critical contributors to cognitive learning gains.

**Conclusion:**

This study underscores the potential of gamified tools in enhancing engagement and knowledge retention, providing a foundation for further integration of gamification into medical education curricula.

## INTRODUCTION

Anatomy is a foundational subject in medical education, serving as the basis for understanding human physiology and clinical practice. However, neuroanatomy, particularly cranial nerve anatomy, presents significant learning challenges for medical students, contributing to a phenomenon known as “neurophobia”- an aversion towards learning neural anatomy due to its complexity.^1^,^2^ Traditional teaching methods in anatomy, such as lectures and textbooks, have been criticized for their lack of engagement and limited retention of knowledge, leading to decreased motivation and poor student outcomes.^3^ To address these limitations, innovative pedagogical approaches like gamification have been introduced to enhance student engagement, motivation and learning outcomes.^4^,^5^

Gamification, defined as the use of game elements in non-game contexts, aims to create immersive and interactive learning experiences that encourage active participation and critical thinking.^6^ In medical education, games have been used not only to introduce new concepts but also to reinforce existing knowledge through activities that offer immediate feedback, motivate learners and facilitate memory retention.^7^,^8^ The use of games like “Anatomy Cluedo”, an adaptation of the popular board game “Cluedo,” is particularly promising for teaching cranial nerve anatomy.

Anatomy Cluedo supports active engagement, intrinsic motivation, and contextualized learning. Flow Theory emphasizes optimal engagement, wherein learners become deeply absorbed in activities that balance challenge and existing skill levels.^4^ Through puzzles, clinical clues, and competitive elements, Anatomy Cluedo aims to foster this state of flow, maintaining student focus and meaningful participation. Similarly, Self-Determination Theory underscores the role of autonomy, competence, and relatedness in promoting intrinsic motivation; the game’s structure enables learners to make decisions, solve anatomical challenges collaboratively, and experience a sense of mastery, thereby enhancing motivation to learn.^9^ Cognitive Load Theory informs the instructional design of the game by emphasizing reduction of extraneous load through structured clues and scaffolded problem-solving while supporting germane load by encouraging active integration of anatomical knowledge.^10^ Overall, this game-based approach transforms traditionally passive learning of cranial nerves into an engaging, clinically contextualized, and competitive experience that enhances learner motivation, knowledge organization, and retention.

While the effectiveness of gamification in improving engagement and motivation is well-documented, it is essential to identify specific game elements that directly contribute to cognitive learning outcomes. By understanding the correlation between engagement features, such as game mechanics, real-time feedback and storytelling and cognitive gains, educators can fine-tune gamified tools like “Anatomy Cluedo” to maximize learning efficiency. Previous studies have demonstrated that specific gamified components can enhance active learning, problem-solving skills and knowledge retention.^6^,^7^,^11^ However, there is a lack of research that quantitatively measures the correlation between these features and cognitive learning outcomes, especially in anatomy education.

This study aimed to evaluate the impact of “Anatomy Cluedo” on learning outcomes among medical students, focusing on the correlation between different gamified elements and cognitive gains.

This study has significant implications for medical education, particularly in teaching complex topics like cranial nerve anatomy. Understanding the correlation between specific gamified features and learning outcomes can inform the design of future game-based educational tools, making them more effective and aligned with pedagogical objectives.^7^. The findings from this study not only validate “Anatomy Cluedo” as an effective educational tool but also contribute to the broader field of educational psychology by providing empirical evidence on the role of gamification in improving cognitive learning outcomes.

## METHODOLOGY

The Anatomy Cluedo game was systematically conceptualized and developed following the AMEE Guide for instrument development to ensure educational rigor and content validity.^12^ The process began with a comprehensive literature review to identify key learning outcomes related to cranial nerve anatomy and effective approaches in game-based learning. Subsequently, a focus group discussion with anatomy faculty and students was conducted to gather contextual insights and learner needs. Findings from the literature and focus group were synthesized to guide item and game-mechanic creation, including clue structure, case vignettes, character roles, and scoring rules. Draft game items and design elements were then developed and reviewed by a panel of subject and educational experts to establish content relevance and clarity (Expert Validation). Cognitive interviews with target-level students were performed to refine game logistics, language clarity, and cognitive demand. A pilot test was subsequently conducted to evaluate gameplay flow, feasibility, and learner engagement. An additional step of constructing validity evidence was integrated by examining alignment between gameplay performance and anatomical knowledge application. Iterative revisions were made at each stage, resulting in a refined, contextually appropriate, and pedagogically grounded game format suitable for quasi-experimental application in anatomy education.

The study was carried out in the Department of Anatomy, Shalamar Medical and Dental College, Lahore, and was completed six months after receiving approval from the review boards. A total of 40 undergraduate medical students^13^,^14^ were recruited as participants for this pilot study. With 20 participants per group, this sample was sufficient to detect large effect sizes, while also providing preliminary data on feasibility, acceptability, and trends in learning outcomes.^15^,^16^ These findings are intended to inform design and sample size calculation of a subsequent, adequately powered trial. Participants were randomly allocated into two equal groups to minimize bias and maintain comparability.

### Ethical considerations:

Ethical approval was obtained from the Institutional Review Board (IRB) of the University of Lahore (ERC139/23/12; dated: December 12, 2023) and Shalamar Medical and Dental College (SMDC-IRB/AL/2024-017; dated March 21, 2024). Written informed consent was obtained from all participants. To ensure confidentiality and anonymity, unique identification codes were used instead of names, and all data were stored in password-protected files accessible only to the research team. Students unwilling to provide informed consent or those with incomplete or inconsistent academic records were excluded to maintain a comparable baseline level of prior knowledge and academic performance across participants, thereby minimizing confounding differences and supporting the reliability of the study results.

### Group-A (Experimental Group):

Played “Anatomy Cluedo. (10 2^nd^ MBBS year and 10 3^rd^ year MBBS students)

### Group-B (Control Group):

Engaged in traditional problem-based learning (PBL). (10 2^nd^ year MBBS and 10 3^rd^ year MBBS students)

### Data collection tools and measures:

Students in both groups completed a pre-test to assess their baseline knowledge of cranial nerve anatomy. Immediately after both cohorts completed their respective interventions (Anatomy Cluedo gameplay for Group A and PBL for Group B), a standardized post-test was administered to objectively evaluate cognitive learning gains. The cognitive gain was calculated as the difference between post-test and pre-test scores. To assess engagement, the Flow Short Scale (FSS) was employed for Group-A during gameplay.^17^ This scale measured parameters such as fluency, absorption, and enjoyment, thereby providing a quantitative estimate of student engagement. The FSS was rated on a Likert scale ranging from one (strongly disagree) to seven (strongly agree), (Annexure 1).

### Statistical Analysis:

In view of small sample size (n = 20 per group) and non-normal distribution of data, within-group analysis (Pre- vs. Post-test) was performed by Wilcoxon signed-rank. The between-group analysis (Learning Gain A vs. B) was undertaken by the Mann–Whitney U test for comparing independent non-parametric data. The difference in mean scores was used to determine statistical significance, with a p-value of less than 0.05 considered significant. In addition, Spearman’s correlation analysis was conducted to examine the relationship between game elements (e.g., mechanics, feedback, and storytelling) and cognitive learning outcomes. Correlation coefficients (r-values) were interpreted as weak (0.0-0.3), moderate (0.3-0.7), or strong (0.7-1.0).^18^ Specific parameters analyzed included student engagement levels, measured by the Flow Short Scale (FSS) scores^17^, and cognitive learning gains, calculated from differences between pre-test and post-test performance.

## RESULTS

The quantitative analysis evaluated the impact of “Anatomy Cluedo” on cognitive learning gains and engagement among medical students. It involved comparing pre-test and post-test scores, analyzing engagement metrics and exploring correlations between game elements and learning outcomes.

### Cognitive Learning Gain:

Group-A improved by an average of 6.4 points indicating that, on average, students learned and improved after the intervention, while Group B’s performance declined by about 3.1 points from pre- to post-test. This yielded a 9.5-point higher learning gain in Group A compared with Group B. The difference between the two groups is not only highly statistically significant (as shown by the Mann–Whitney U test, p < 0.001) but also educationally meaningful, reflecting a substantial improvement in cognitive learning performance for Group-A relative to Group-B, underscoring the effectiveness of “Anatomy Cluedo” in improving knowledge retention. The results are summarized in Table-I.

**Table-I:** Pre-test and Post-test Scores with Cognitive Learning Gain.

Group	Pre-test Score (Mean ± SD)	Post-test Score (Mean ± SD)	Cognitive Learning Gain (Mean ± SD)	Statistical Significance (p-value)
Group-A (Anatomy Cluedo)	34.7 ± 7.2	41.1 ± 3.3	6.4 ± 5.4	< 0.001
Group-B (PBL)	26.4 ± 7.0	23.3 ± 9.9	-3.1 ± 8.0	

All values represent percentages and are presented as Mean ± SD. Cognitive learning gain was calculated as post-test minus pre-test scores. An Independent-Samples Mann–Whitney U test was used to compare learning gains between groups. A p-value < 0.05 was considered statistically significant.

### Engagement Metrics:

The FSS results indicated high engagement levels among Group-A students, the mean scores for each dimension are presented in Table-II. “Anatomy Cluedo” successfully engaged students, with high scores across all three dimensions of the Flow Short Scale, indicating an immersive and enjoyable learning experience Table-II.

**Table-II:** Engagement Metrics in Group-A (Flow Short Scale).

Engagement Dimension	Mean Score (out of 5)	Interpretation
Fluency	4.1	High level of uninterrupted learning flow
Absorption	4.3	Deep immersion in gameplay
Enjoyment	4.6	High level of satisfaction and fun

### Correlation Analysis:

Spearman’s correlation analysis was conducted immediately after the post-test to examine bivariate associations between engagement variables (independent variables) and cognitive learning gains (dependent variable). A strong positive correlation was observed between overall engagement score and learning gains (r = 0.78, p < 0.01). Game mechanics (r = 0.74, p < 0.01), feedback mechanisms (r = 0.70, p < 0.01), and fluency/absorption (r = 0.72, p < 0.01) also showed strong positive correlations with cognitive gains. Storytelling demonstrated a moderate positive correlation (r = 0.67, p < 0.05). These results indicate that higher engagement with key game elements consistently aligned with greater learning improvements (Table-III).

**Table-III:** Correlation Analysis of Game Elements with Cognitive Learning Gains.

Independent Variables	Spearman’s r	p-value
Overall Engagement	0.78	< 0.01
Game Mechanics	0.74	< 0.01
Feedback Mechanism	0.70	< 0.01
Storytelling	0.67	< 0.05
Fluency and absorption	0.72	< 0.01

Positive r-values indicate that higher engagement and favorable game-element ratings were associated with greater cognitive learning gain.

### Multivariate analysis:

A multiple regression analysis was conducted to examine whether game design elements predicted cognitive learning gain. The overall regression model was statistically significant, F (5, 34) = 11.4, p < 0.001, explaining 77% of the variance in learning gain (Adjusted R² = 0.73). Overall Engagement (β = 0.39, p = 0.002), Game Mechanics (β = 0.28, p = 0.007), Feedback Mechanism (β = 0.20, p = 0.040), and Fluency & Absorption (β = 0.24, p = 0.020) were significant positive predictors, indicating that higher levels of these factors were associated with greater cognitive learning gains. Storytelling (β = 0.13, p = 0.110) contributed positively but not significantly when other predictors were controlled (Table-IV). These findings suggest that engagement and well-structured game mechanics are key components driving effective learning outcomes in the gamified intervention.

**Table-IV:** Multiple Regression Analysis Predicting Cognitive Learning Gain from Game Elements.

Predictor Variable	B	SE B	β	t	p-value
Overall Engagement	0.42	0.12	0.39	3.45	0.002
Game Mechanics	0.31	0.11	0.28	2.84	0.007
Feedback Mechanism	0.22	0.10	0.20	2.10	0.040
Storytelling	0.15	0.09	0.13	1.67	0.110
Fluency & Absorption	0.27	0.11	0.24	2.36	0.020
Constant	0.85	1.16	—	0.73	0.47

## DISCUSSION

The results of this study demonstrate that Anatomy Cluedo, a serious game–based learning tool, effectively enhanced cognitive learning and engagement in cranial nerve anatomy. These findings align with existing evidence showing that gamification strategies improve learning outcomes, motivation, and learner interaction in medical education.^7^,^11^,^19^ Importantly, this study extends the literature by directly comparing a serious game format with a traditional small-group/problem-based learning (PBL) approach—an area where prior research remains limited. Similar to our findings, O’Leary et al. reported that game-based learning produced greater short-term knowledge gains than PBL in clinical teaching contexts^20^, while Almodaires et al. and Azer also observed higher engagement and retention in game-supported small-group teaching compared to conventional formats 21,22 These results suggest that integrating structured game mechanics with case-based learning can address limitations of passive or discussion-dependent formats and enhance flow-related engagement, sustained attention, and knowledge consolidation. Overall, our findings support a growing body of literature advocating serious games as an effective complement or alternative to traditional small group learning models in anatomy education.

### Cognitive learning gains:

The results indicated a significant increase in cognitive learning gains among students who played “Anatomy Cluedo” compared to those who engaged in PBL (Table-I). Group-A’s mean cognitive gain (33%) was substantially higher than that of Group-B (19%). The improvement in knowledge retention aligns with previous studies demonstrating the positive impact of gamification in medical education.^23^,^24^ Gamified approaches, by providing active learning experiences, can reduce cognitive load and facilitate better understanding of complex anatomical concepts.^10^

**Fig.1:**
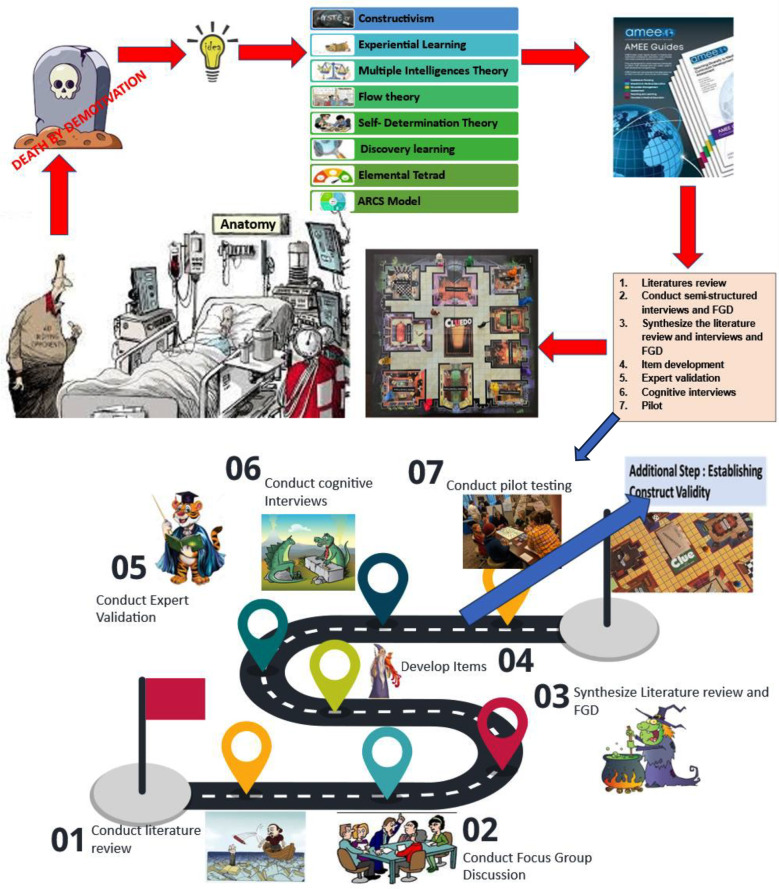
This illustration represents the structured development of anatomy education, designed to address the challenges posed by demotivated and disengaged students in traditional teaching settings. The process based on educational theories supporting gamification follows the AMEE Guide 87 seven-step approach, ensuring an evidence-based and student-centered methodology.

The significant increase in post-test scores for Group-A suggests that game-based learning creates a more engaging environment that supports long-term memory formation. This was evident in the substantial cognitive gains observed in Group-A, reinforcing the argument that games like “Anatomy Cluedo” can be effective tools for teaching complex subjects like neuroanatomy.

### Engagement and learning outcomes:

The strong correlation between engagement metrics and cognitive learning gains (r = 0.78) suggests that increased engagement positively influences learning outcomes, a finding consistent with existing literature on gamification in education.^25^,^26^

### Role of Game Elements in Cognitive Learning:

The correlation analysis provided insights into how specific game elements impact cognitive learning. Key findings include:

### Game Mechanics:

O’Leary et al. reported that structured game mechanics in medical education promote deeper cognitive processing by guiding learners through progressive problem-solving challenges and reinforcing knowledge application^20^. Similarly, Azer highlighted that clearly designed rules and goal-oriented play enhance student focus and retention by reducing ambiguity in learning tasks.^2^,^21^ In line with these findings, the present study demonstrated a strong positive correlation between game mechanics and cognitive learning gains (r = 0.74, p < 0.01), suggesting that well-defined rules and structured gameplay within Anatomy Cluedo contributed meaningfully to knowledge retention and effective learning.^20^,^21^

### Feedback Mechanism:

The significant positive correlation (r = 0.70, p < 0.01) between real-time feedback and cognitive gains supports the idea that immediate feedback reinforces correct knowledge and rectifies misconceptions promptly 7. This finding is consistent with educational theories emphasizing feedback’s role in learning, particularly in complex subjects like anatomy.^27^

### Storytelling:

The moderate correlation between storytelling and cognitive gains (r = 0.67, p < 0.05) suggests that narrative elements help contextualize anatomical concepts, making them more relatable and memorable 8. Storytelling in “Anatomy Cluedo” allowed students to connect clinical scenarios with anatomical knowledge, aligning with studies highlighting the benefits of narrative-based learning in medical education.^28^

### Implications for Medical Education:

The findings of this study have significant implications for medical education, particularly for teaching complex topics such as cranial nerve anatomy. The strong correlations between engagement, key game elements, and cognitive learning gains support the effectiveness of game-based learning approaches in enhancing student understanding and participation. “Anatomy Cluedo” as an effective educational tool. Gamification, by integrating interactive and immersive elements, can address traditional learning methods’ limitations, making it a promising approach for medical curricula. 4

The success of “Anatomy Cluedo” suggests that game-based learning tools can be adapted to teach other complex subjects in medical education, such as physiology, pathology and pharmacology. Future research could explore the scalability of such tools, including digital adaptations, to broaden their accessibility and impact.

### Limitations

While the study demonstrates the effectiveness of Anatomy Cluedo, certain limitations should be acknowledged. The sample size was relatively small, which may limit the generalizability of the findings. Although a control group was included, the Hawthorne effect may still have influenced participant behavior, as students were aware they were part of an innovative learning activity. Future studies should consider additional strategies to minimize this effect, such as blinding participants to the comparative nature of the study or using repeated exposure to normalize the intervention.

## CONCLUSION

The results of this study underscore the potential of game-based learning tools such as Anatomy Cluedo in enhancing cognitive learning and engagement among medical students. Beyond demonstrating effectiveness, a key novelty of this work lies in examining how specific gamification components relate to learning outcomes, thereby offering deeper insight into which elements of gameplay contribute most to cognitive gains. By providing a structured, interactive, and feedback-rich environment, Anatomy Cluedo addresses the challenges of teaching complex anatomical content such as cranial nerves. This study contributes to the expanding body of literature on gamification in medical education and introduces an evidence-based framework for understanding the mechanisms through which game elements enhance learning, paving the way for future innovations in educational design.

### Future recommendations:

Furthermore, the current study focused exclusively on cranial nerve anatomy; therefore, future research should evaluate the application of Cluedo-based learning across other anatomy topics and basic science disciplines to determine its broader educational utility. Additional studies may also explore digital adaptations to scale the intervention and assess its impact across diverse learner groups.

## ANNEXURE-1

***Flow Short Scale:*** The Flow Short Scale (FSS) is used to assess flow experience as a state variable. The FSS has demonstrated construct validity and good psychometric properties (α=.90), and it measures a stable three-factor structure comprising fluency of performance, absorption by activity, and perceived importance or outcome importance of the activity^17^.

**Components of Flow experience**

***The key elements that contribute to the flow experience, a state of optimal engagement and immersion in an activity*.**

***Test construction:*** The FSS consists of a total of 13 items. Items 1-10 assess components of the flow experience. These 10 items can be subdivided into two subscales (1) Fluency (items: 2, 4, 5, 7, 8, 9) and (2) Absorption (items: 1, 3, 6, 10). In addition to the flow assessment itself, items 11-13 measure a Worry component. These 13 items are to be answered on a seven-point Likert-scale from “strongly disagree” to “strongly agree.”

**Scoring.**

*The scoring parameters utilized in the Flow Short Scale (FSS), a tool designed to measure the flow experience during an activity*.

***Interpretation:***

**Very high flow total** scores mean that both Fluency and Absorption are high. Medium values may mean that both subscales are medium high.
